# Alpha-Mangostin Alleviates Mitochondrial Damage and Autophagy Dysregulation in the MPP^+^ Cellular Model of Parkinson's Disease

**DOI:** 10.1155/adpp/5567858

**Published:** 2025-08-18

**Authors:** Korede Jacob Abraham, Permphan Dharmasaroja

**Affiliations:** Department of Anatomy, Faculty of Science, Mahidol University, Bangkok, Thailand

**Keywords:** alpha-mangostin, autophagy, mitochondria, MPP^+^, mTOR, p70S6K

## Abstract

Alpha-mangostin (α-M), a xanthone derivative with known antioxidative properties, has demonstrated a protective effect on neurons under oxidative stress, a key factor in the pathogenesis of Parkinson's disease (PD). However, its impact on mitochondrial integrity and autophagy in PD remains insufficiently understood. Therefore, the present study aimed to investigate the role of α-M in regulating defective mitochondrial proteins and its influence on the mTOR pathway, both of which are critical in the regulation of autophagy. This study investigated the effects of α-M pretreatment on 1-methyl-4-phenylpyridinium (MPP^+^)-induced neurotoxicity in SH-SY5Y dopaminergic neurons. MPP^+^, a mitochondrial complex I inhibitor, significantly reduced the expression of mitochondrial proteins NDUFS3 and TIMM23, induced mitochondrial damage, and triggered excessive autophagy, as evidenced by elevated LC3-II/LC3-I ratio and phospho-Beclin-1 expression. These changes were accompanied by dysregulation of the mTOR signaling pathway, including increased phosphorylation of mTOR and suppression of its downstream effector p70S6K. α-M pretreatment restored NDUFS3 and TIMM23 levels, preserved mitochondrial morphology and membrane potential, and reduced autophagy activation by mitigating MPP^+^-induced LC3B accumulation and Beclin-1 activation. Additionally, α-M restored balance in the mTOR signaling pathway by reducing mTOR phosphorylation and restoring p70S6K activity, counteracting the autophagic dysregulation caused by MPP^+^. Importantly, α-M exhibited no toxicity under normal conditions, indicating its protective effects are context-dependent and activated only during cellular stress. These findings highlight the potential of α-M as a therapeutic agent for PD, providing neuroprotection through its targeted modulation of mitochondrial proteins and mTOR signaling that regulates autophagy.

## 1. Introduction

Parkinson's disease (PD) is characterized by the progressive depletion of dopaminergic neurons in the substantia nigra pars compacta and the emergence of intraneuronal protein inclusions containing abnormal aggregations of alphasynuclein, ultimately resulting in movement disorders [1]. The clinical manifestation of PD includes motor symptoms such as rest tremors, bradykinesia, and muscle rigidity, along with associated non-motor symptoms like depression and sleep disorders [2].

The underlying molecular pathogenesis of PD is linked to mitochondrial dysfunction, oxidative stress, and the initiation of the apoptotic cascade [3]. The active metabolite 1-methyl-4-phenylpyridinium ion (MPP^+^) derived from 1-methyl-4-phenyl-1,2,3,6-tetrahydropyridine (MPTP) serves as a critical element in modeling PD. MPP^+^ induces a PD-like pathology in animals and cellular models by robustly inhibiting complex I of the mitochondrial electron transport chain, along with an increase in reactive oxygen species (ROS) levels [4, 5]. The apoptotic cascade is activated during MPP^+^-induced cell death through alterations in mitochondrial membrane permeability and the controlled release of cytochrome c from mitochondria, subsequently activating caspase-3, leading to nuclear DNA condensation, fragmentation, and ultimately, apoptosis [6, 7]. Similar apoptotic and autophagic mechanisms, mediated through caspase-3 and Beclin-1 activation, have been observed in response to environmental toxicants such as microplastics, emphasizing the broader relevance of oxidative stress and NF-κB/Nrf2 signaling in neurotoxicity [8].

MPP^+^ also induces elevated expressions of microtubule-associated protein light-chain 3 (LC3-II/LC3-I) and Beclin-1, serving as markers of autophagy, in SH-SY5Y cells [8, 9]. Autophagy, a homeostatic process involving the degradation of internal components in the cytoplasm, plays a pivotal role in cellular health, eliminating damaged organelles, misfolded proteins, intracellular pathogens, and oversized targets unsuitable for degradation by other systems [9, 10]. Deficiencies in the degradation process, particularly in the clearance of mutant α-synuclein, have been implicated in PD [10, 11]. The regulation of autophagy involves various signaling pathways, including phosphatidylinositol 3-kinase (PI3K)/protein kinase B (Akt)/mammalian target of rapamycin (mTOR), Jun N-terminal kinase (JNK), Janus kinases (JAK)/signal transducer and activator of transcription proteins (STAT), and MAPK/NF-κB/MLKL signaling cascades, which are increasingly recognized in the context of neuroinflammation and neurodegeneration [12–15]. In addition, our group has demonstrated that MPP^+^ suppresses mitochondrial membrane proteins TIMM23 and NDUFS3 [16]. TIMM23 plays a crucial role in mediating the translocation of transit peptide-containing proteins across the inner mitochondrial membrane [17]. NDUFS3, a core subunit of NADH dehydrogenase (complex I), is recruited to the inner mitochondrial membrane, triggering a programmed cell death pathway that leads to mitochondrial dysfunction and the generation of ROS [18].

Various antioxidants, such as xanthones, have shown a protective effect on neurons facing oxidative stress conditions [19–21]. The fruit hull of mangosteen (*Garcinia mangostana* Linn.) is known for its antioxidative properties. Within the fruit hull, multiple xanthone derivatives, including alpha-mangostin (α-M), contribute to this antioxidative effect. α-M is a polyphenolic xanthone derivative from mangosteen. The xanthone structure of α-M suggests a robust antioxidant action. Moreover, α-M is recognized for its anti-inflammatory, antibacterial, and anticancer effects [22]. Potential roles of α-M in protection against neuroinflammation and neurotoxicity have also been reported [23–25].

Reportedly, studies showed that α-M exerts a neuroprotective effect in models of PD. In SH-SY5Y cells, we have shown that cotreatment of α-M with MPP^+^ reduced MPP^+^-induced cell death, ROS formation, and modulated pro- and antiapoptotic gene expression, suppressing caspase-3 activation [26]. In rotenone-induced cytotoxicity, α-M mitigated alpha-synuclein (α-Syn) aggregation and prevented tyrosine hydroxylase (TH) loss. Additionally, it reversed rotenone-induced ROS overproduction, caspase-8/-3 activation, and mitochondrial dysfunction [27]. The mechanistic study unveiled that α-M functions by inhibiting nuclear factor kappa B (NF-kappaB) and NADPH oxidase. Moreover, α-M showed protective effects against both alpha-synuclein-induced microglial activation and direct neurotoxicity [28]. In an in vivo rat model of PD, chronic rotenone treatment induced oxidative stress, TH-positive dopaminergic neuron loss, and motor deficits. α-M restored antioxidant enzyme levels, improved locomotor activity and memory, and reduced phosphorylated alpha-synuclein [29]. 5′AMP-activated protein kinase (AMPK) is recognized for its ability to trigger autophagy, leading to the subsequent elimination of α-Syn aggregates. A study showed that α-M provides neuroprotection against rotenone-induced α-Syn accumulation in the mouse striatum and cortex. This protection is achieved by activating AMPK and upregulating markers associated with autophagy (LC3-II/LC3-I and Beclin-1) and lysosomal function (TFEB) [30].

As there is insufficient understanding of the effect of α-M on the mitochondrial integrity in PD, the present study aimed to study defective mitochondrial proteins in an in vitro model of PD. Our initial focus was on exploring the impact of α-M pretreatment on MPP^+^-induced neurotoxicity in SH-SY5Y cells differentiated into a neuronal phenotype. We specifically investigated the influence on the mTOR pathway and its downstream proteins, namely p70S6K and 4E-BP1, which play crucial roles in regulating mitophagy. Subsequently, we assessed the presence of defective mitochondria by examining the localization of LC3-B puncta and evaluating the expression of mitochondrial membrane proteins, including NDUFS3 and TIMM23.

## 2. Materials and Methods

### 2.1. SH-SY5Y Cell Culture and Differentiation

SH-SY5Y human neuroblastoma cells were initially grown in T-25 flasks and subcultured in a variety of plates suitable for the experiment. The culture medium consisted of minimum essential medium (MEM) and Ham's F-12 nutrient mixture, supplemented with 10% heat-inactivated fetal bovine serum (FBS), 1 mM sodium pyruvate, nonessential amino acids, and 7.5% sodium bicarbonate. Cells were subsequently scaled up in T-75 flasks until they reached 70%–90% confluence, as confirmed under an inverted microscope. At this stage, cells were seeded into appropriate culture plates for differentiation and treatment. Undifferentiated SH-SY5Y cells were subcultured from the confluent flasks, plated into the respective culture plates, and allowed to acclimatize for 24 h. Differentiation was induced with 10 μM all-*trans* retinoic acid (RA) in growth media containing 1% heat-inactivated FBS. The media was refreshed every 72 h, and the treatment continued for 10 days. Thereafter, cells were treated with α-M (≥ 98% purity level tested using HPLC; Sigma-Aldrich, St. Louis, MO, USA) or MPP^+^ (Sigma-Aldrich), according to the experiment design.

### 2.2. MTT Assay

Cell viability was assessed using the MTT [3-(4,5-dimethylthiazol-2-yl)-2,5-diphenyl tetrazolium bromide] assay. SH-SY5Y cells were seeded in a 96-well plate at 1 × 10^4^ cells/well and incubated for 24 h at 37°C in 5% CO_2_. Thereafter, cells were incubated with fresh media containing 0.5 mg/mL MTT (Sigma-Aldrich). The plates were kept to protect them from light. After 3 h of incubation, the MTT-containing media were removed, and dimethyl sulfoxide (DMSO) was added to dissolve the formazan crystals. The absorbance was measured using a microplate reader (BioTek, Vermont, USA), and the data were expressed as a percentage relative to the untreated control.

### 2.3. Western Blot Analysis

SH-SY5Y cells were seeded in a 6-well plate at a density of 3 × 10^5^ cells/well and treated under the specified experimental conditions. Following treatment, cells were washed with PBS, trypsinized, centrifuged, and resuspended in cold PBS. The cell pellets were washed and lysed in radioimmunoprecipitation assay (RIPA) buffer supplemented with 1% protease inhibitor cocktail. The lysates were centrifuged at 14,000 rpm for 15 min at 4°C, and the supernatants were collected and stored at −80°C until further use. Protein samples were prepared by mixing the supernatant with the sample buffer. Proteins (30 μg per sample) were separated using SDS-PAGE and transferred to polyvinylidene fluoride (PVDF) membranes. The membranes were blocked with 5% skimmed milk in Tris-buffered saline (TBST with 0.1% Tween-20) and incubated overnight at 4°C with primary antibodies. These included mouse anti-TH at a dilution of 1:1000, rabbit anti-phospho-Akt at 1:1000, rabbit Akt at 1:1000, rabbit anti-phospho-p70S6 at 1:1000, rabbit anti-p70S6 at 1:1000, rabbit anti-phospho-4E-BP1 at 1:1000, rabbit 4E-BP1 at 1:1000, rabbit anti-phospho-mTOR at 1:1000, rabbit anti-mTOR at 1:1000, rabbit anti-TIMM23 at 1:1000, and rabbit anti-NDUFS3 at 1:1000 (all purchased from Abcam, Cambridge, UK). Rabbit anti-phospho-Beclin-1 (Ser15; 1:1000) was obtained from Cell Signaling (MA, USA). Additionally, rabbit anti-LC3-B (1:1000) and mouse anti-β-actin (1:5000) were purchased from Sigma-Aldrich (MO, USA). After primary antibody incubation, the membranes were washed with TBST and incubated with horseradish peroxidase (HRP)-conjugated secondary antibodies (anti-rabbit IgG or anti-mouse IgG) (Cell Signaling, MA, USA) at a dilution of 1:1000. Protein bands were detected using the ECL chemiluminescence system (Thermo Fisher Scientific, MA, USA). The band intensity was measured using ImageJ software (National Institutes of Health, Maryland, USA).

### 2.4. Apoptotic Nuclear Staining

Following the completion of the experimental treatment conditions, SH-SY5Y cells were washed with PBS and fixed with 4% paraformaldehyde. Subsequently, cells were washed thrice in cold PBS for 5 min each. Staining was performed using 10 μg/mL Hoechst 33,258 (Abcam) in the dark for 10 min, followed by three additional washes with PBS, each lasting 5 min. The stained coverslips were then mounted, and nuclear morphology was visualized using a confocal microscope (Olympus FV10i, Tokyo, Japan).

### 2.5. Mitochondrial Staining

SH-SY5Y cells were plated in 24-well plates and incubated with 50 nM MitoTracker Red (Cell Signaling) in culture media for 15 min in the dark. Following incubation, cells were washed thrice with 1X PBS and permeabilized with 0.25% Triton X-100. The stained cells were then visualized using a confocal microscope (Olympus FV10i), and the fluorescence intensity was analyzed using ImageJ software.

### 2.6. Immunocytochemistry

Following treatment, cells were fixed with 4% paraformaldehyde for 10–15 min, washed with PBS, and permeabilized using 0.25% Triton X-100. Nonspecific binding was blocked with 10% normal goat serum or 1% BSA in PBST, depending on the protocol. The cells were incubated overnight at 4°C with primary antibodies, including anti-LC3-B (1:200; Sigma-Aldrich, MO, USA), TIMM23 (1:700), and NDUFS3 (1:700). After washing with PBS or PBST, they were incubated for 1 h with Alexa Fluor 488-coupled goat antirabbit IgG secondary antibody (Cell Signaling) in the dark. Nuclear counterstaining was performed with Hoechst 33,258. The coverslips were mounted using antifade mounting medium, covered with aluminum foil, and stored at 4°C. Fluorescence was visualized using a confocal microscope (Olympus FV10i or BX-53), and images were analyzed using ImageJ software. For fluorescent intensity analysis of TIMM23 and NDUFS3, regions of interest (ROI) were selected in ImageJ from four different areas per sample, with mean gray values calculated for three independent samples per group.

### 2.7. Mitochondrial Membrane Potential Assay

SH-SY5Y cells were seeded in 6-well plates at a density of 2 × 10^4^ cells/well in 2 mL of medium and incubated for 24 h at 37°C in a humidified incubator with 5% CO_2_. Following treatment, the cells were trypsinized and centrifuged at 300 rpm for 5 min. The resulting pellet was washed twice with cold PBS and resuspended in 500 μL of JC-10 dye solution (JC-10 Mitochondrial Membrane Potential Assay Kit; Abcam) at a concentration of 3 × 10^5^ cells/tube. The suspension was incubated for 30 min at 37°C in 5% CO_2_ protected from light. Mitochondrial membrane potential was then assessed using a flow cytometer, with fluorescence measured at an excitation wavelength of 488 nm and emission wavelengths of 530 and 585 nm.

### 2.8. Statistical Analysis

Results from multiple experiments were presented as the mean ± standard error of the mean (SEM) or standard deviation (SD). Statistical significance was assessed using one-way analysis of variance (ANOVA) followed by Tukey's multiple comparison test. A *p*-value of < 0.05 was considered statistically significant. All analyses were performed using GraphPad Prism Version 9 (CA, USA).

## 3. Results

### 3.1. Increased Expression of TH in Differentiated SH-SY5Y Cells

**Figure 1:**
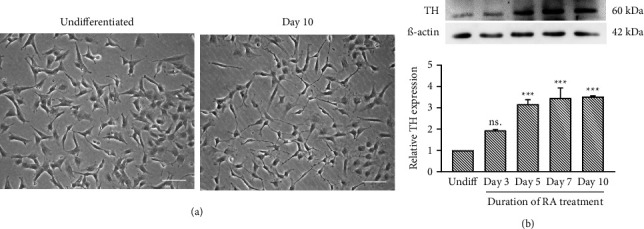
Differentiation of SH-SY5Y cells induced with 10 μM RA into a neuronal phenotype. (a) An inverted microscope revealed the morphology of undifferentiated cells and 10-day RA-induced differentiated cells. Scale bar = 40 μm. (b) Western blotting analysis and quantification of TH in SH-SY5Y cells treated with 10 μM RA for 3, 5, 7, and 10 days. Values were normalized to the control group (set as 1). Data were expressed as mean ± SEM (*n* = 3). ^∗∗∗^*p* < 0.001; ns., not significant compared with the undifferentiated group.

To evaluate the effects of α-M in neuronal cells, SH-SY5Y cells were differentiated into a neuronal phenotype by incubating in 10 μM of RA with 1% FBS for 10 days. Differentiated cells clearly showed multipolar cell bodies with increased and elongated neuritic outgrowths, while undifferentiated cells showed shorter neuritic processes and neuroblast-like morphology (Figure 1(a)). To support the neuronal differentiation, the expression of TH, a marker for mature dopaminergic neurons and the rate-limiting enzyme in the biosynthesis of dopamine, was examined. Western blot analysis showed a significantly increased TH expression from Day 5 to Day 10 of RA differentiation (*p* < 0.001; Figure 1(b)). This protocol was used to generate mature neuronal cells for subsequent experiments.

### 3.2. Effects of α-M Pretreatment on the Survival of MPP^+^-Treated Neuronal Cells

**Figure 2:**
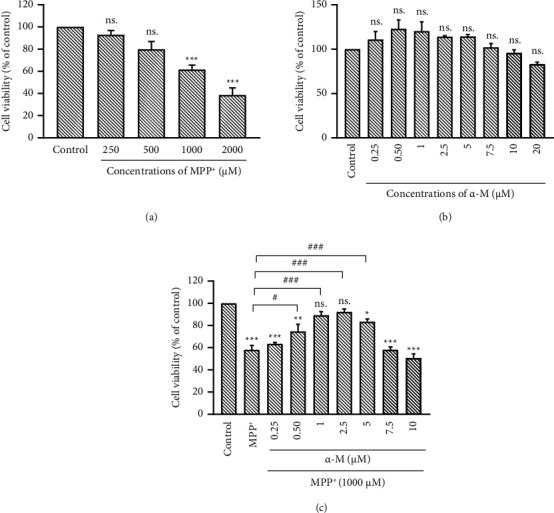
Effects of α-M pretreatment on the survival of MPP^+^-treated neuronal cells. Cell viability of 10-day RA-induced differentiated SH-SY5Y cells was assessed using an MTT assay. (a) Treatment with 250, 500, 1000, and 2000 μM of MPP^+^ for 24 h. (b) Treatment with α-M at concentrations ranging from 0.25 to 20 μM for 1 h. (c) α-M pretreatment in MPP^+^-treated neuronal cells. Cells were treated with α-M for 1 h, followed by exposure to 1000 μM MPP^+^ for 24 h. Data were expressed as mean ± SEM (*n* = 3). ^∗^*p* < 0.05; ^∗∗^*p* < 0.01; ^∗∗∗^*p* < 0.001; ns., not significant compared with the control group. ^#^*p* < 0.05; ^###^ *p* < 0.001.

First, we determined the concentration of MPP^+^ for the generation of a PD model. Differentiated SH-SY5Y cells were treated with various concentrations of MPP^+^ for 24 h, and cell survival was assessed using an MTT assay. The treatment with MPP^+^ at concentrations of 250, 500, 1000, and 2000 μM for 24 h resulted in a significant decrease in cell viability. However, the concentration of 1000 μM was selected for further experiments since it induced a 40% reduction in cell viability (*p* < 0.001 compared with control; Figure 2(a)). Next, we examined the effect of α-M. Treatment with α-M at concentrations ranging from 0.25 to 20 μM for 1 h showed no significant impact on the cell viability of neuronal cells (Figure 2(b)). Then, we examined the effect of α-M pretreatment in MPP^+^-treated neuronal cells. Pretreatment with α-M for 1 h, followed by exposure to 1000-μM MPP^+^ for 24 h, revealed that concentrations of 0.5, 1, 2.5, and 5 μM α-M reduced neuronal death compared with those treated with MPP^+^ alone (*p* > 0.05 for 0.5 μM and *p* > 0.001 for 1, 2.5 and 5 μM of α-M; Figure 2(c)). Higher concentrations of α-M did not improve cell viability. Thus, the experimental setting of a 1 h pretreatment with 5 μM α-M followed by exposure to 1000 μM MPP^+^ for 24 h was used for subsequent experiments.

### 3.3. Effects of α-M Pretreatment in the mTOR Pathway

**Figure 3:**
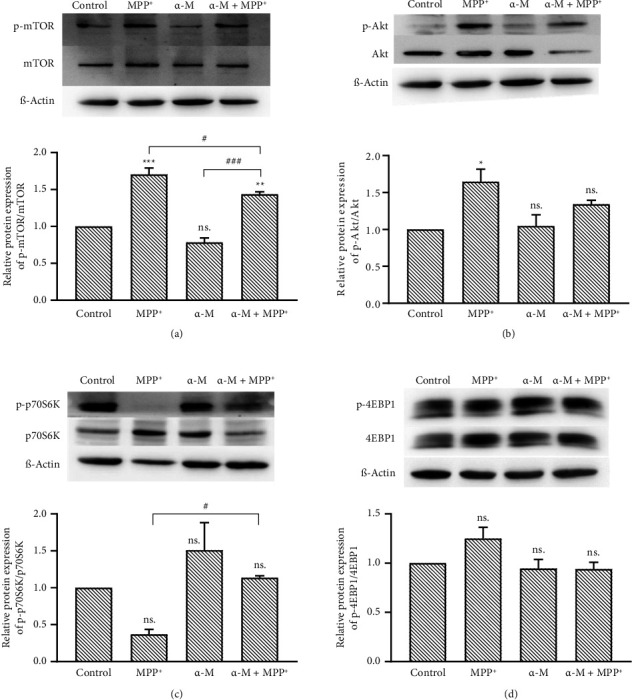
Western blotting analysis of mTOR, Akt, p70S6K, and 4EBP1 expression in MPP^+^-treated differentiated SH-SY5Y cells pretreated with 5 μM α-M. (a) Representative blots of mTOR and p-mTOR and quantification of the relative ratio of p-mTOR to total mTOR. (b) Representative blots of Akt and *p*-Akt and quantification of the relative ratio of p-Akt to total Akt. (c) Representative blots of p70S6K and p-p70S6K and quantification of the relative ratio of p-p70S6K to total p70S6K. (d) Representative blots of 4EBP1 and p-4EBP1 and quantification of the relative ratio of p-4EBP1 to total 4EBP1. The band density was normalized to β-actin. Values were normalized to the control group (set as 1). Data represent mean ± SEM (*n* = 3). ^∗^*p* < 0.05; ^∗∗^*p* < 0.01; ^∗∗∗^*p* < 0.001; ns., not significant compared with the control group. ^#^*p* < 0.05; ^###^*p* < 0.001.

Protein expression of the mTOR pathway was evaluated through Western blot analysis. Treatment with 1000 μM MPP^+^ markedly elevated mTOR phosphorylation (p-mTOR) in comparison to the control, whereas 5 μM α-M did not influence p-mTOR. Notably, in cells subjected to pretreatment with α-M followed by exposure to MPP^+^, the expression of p-mTOR was significantly diminished relative to cells without α-M pretreatment (*p* < 0.05; Figure 3(a)). Investigating the expression of Akt, an upstream molecule of mTOR, revealed that MPP^+^ induced phosphorylation of Akt (p-Akt), while α-M exhibited no effect when compared with the control. However, the pretreatment of α-M followed by MPP^+^ did not significantly alter p-Akt compared with MPP^+^ alone, despite a slight increase being observed (Figure 3(b)).

We further assessed the activation of p70S6K and 4E-BP1, the downstream molecules of the mTOR pathway. MPP^+^ exhibited a nonsignificant decrease in the phosphorylation of p70S6K (p-p70S6K) compared with the control, whereas α-M demonstrated an increase (Figure 3(c)). However, the pretreatment of α-M followed by MPP^+^ significantly elevated p-p70S6K compared with MPP^+^ alone (*p* < 0.05). In the case of phosphorylated-4EBP1 (p-4EBP1), MPP^+^ showed a nonsignificant increase in its expression compared with the control, while α-M displayed no discernible effect. However, the α-M pretreatment followed by MPP^+^ resulted in a decreased phosphorylation of p-4EBP1 compared with MPP^+^ alone, although this reduction was not statistically significant (Figure 3(d)). Overall, the results unequivocally indicate that, in cells exposed to MPP^+^, the pretreatment with α-M suppresses the phosphorylation of mTOR but concurrently enhances the downstream phosphorylation of p70S6K, opposing the effect of MPP^+^.

### 3.4. Effects of α-M Pretreatment on the Formation of the Mitophagosome

**Figure 4:**
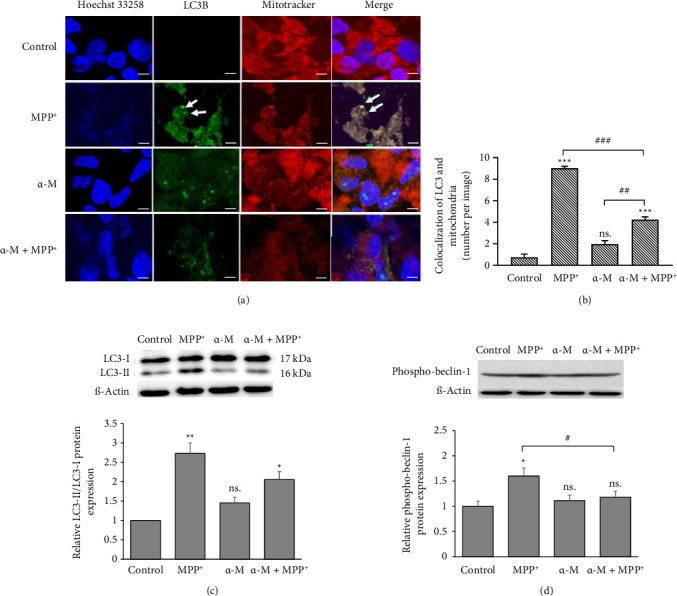
Effect of α-M pretreatment on mitophagy in MPP^+^-treated differentiated SH-SY5Y cells. (a) Immunofluorescence images of MitoTracker Red and LC3B (green) to visualize mitochondria and mitophagy, respectively. White arrows indicate LC3B puncta and colocalization of mitochondria and LC3B. Scale bar = 5 μm. (b) Quantification of colocalization of MitoTracker Red and LC3B. (c) Representative blots of LC3-I and LC3-II and quantification of the relative ratio of LC3-II to LC3-I. (d) Representative blots and quantification of phosphor-Beclin-1. Expression values were normalized to the control group (set as 1). Data were expressed as mean ± SEM (*n* = 3). ^∗∗∗^*p* < 0.001; ns., not significant compared with the control group. ^##^*p* < 0.01; ^###^*p* < 0.001.

To assess the formation of the mitophagosome, we examined the colocalization of LC3B and MitoTracker. Qualitative observations revealed that immunofluorescence staining of the LC3B protein displayed a greater number of LC3B puncta in the MPP^+^-treated group, which had areas of mitochondria labeled using MitoTracker Red, compared with the control group (Figure 4(a)). Conversely, cells subjected to α-M pretreatment followed by MPP^+^ exhibited a reduced number of LC3B puncta colocalized with areas of mitochondria compared with cells without α-M pretreatment. Quantitatively, the calculation of the number of LC3B puncta that colocalized with MitoTracker Red-stained mitochondria was performed per cell. MPP^+^ significantly increased the number of LC3B puncta (*p* < 0.001), whereas α-M showed a nonsignificant increase compared with the control (Figure 4(b)). However, α-M pretreatment followed by MPP^+^ significantly decreased the number of LC3B puncta compared with MPP^+^ alone (*p* < 0.001). The changes in LC3B protein expression were assessed by Western blot analysis (Figure 4(c)). The results demonstrated a significant increase in the conversion of LC3-I to LC3-II, indicated by the LC3-II/LC3-I ratio, in the MPP^+^-treated group compared with the control (*p* < 0.01). This increase was attenuated in the group pretreated with α-M. A similar trend was observed for phospho-Beclin1 expression, which was significantly reduced in the MPP^+^ group following α-M pretreatment compared with MPP^+^ alone (*p* < 0.05; Figure 4(d)).

### 3.5. Effects of α-M Pretreatment on the Expression of Mitochondrial Proteins NDUFS3 and TIMM23

**Figure 5:**
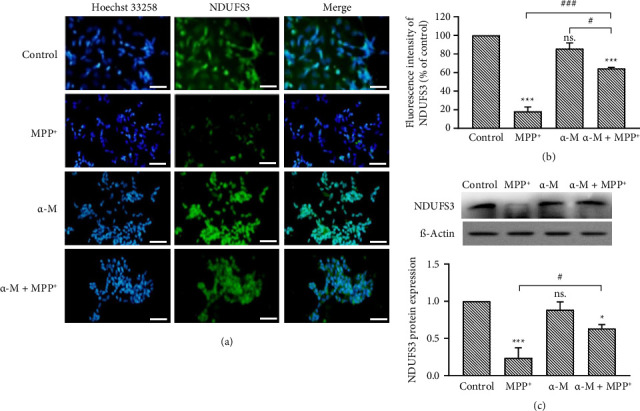
Expression of mitochondrial protein NDUFS3 in MPP^+^-treated differentiated SH-SY5Y cells pretreated with α-M. (a) Immunofluorescence images of NDUFS3 (green). Scale bar = 50 μm. (b) Quantification of NDUFS3 immunofluorescence intensity. Values represent the mean percentage of the control ± SEM (*n* = 3). (c) Western blotting analysis and quantification of NDUFS3. Expression values were normalized to the control group (set as 1). Data were expressed as mean ± SEM (*n* = 3). ^∗^*p* < 0.05; ^∗∗∗^*p* < 0.001; ns., not significant compared with the control group. ^#^*p* < 0.05; ^###^*p* < 0.001.

**Figure 6:**
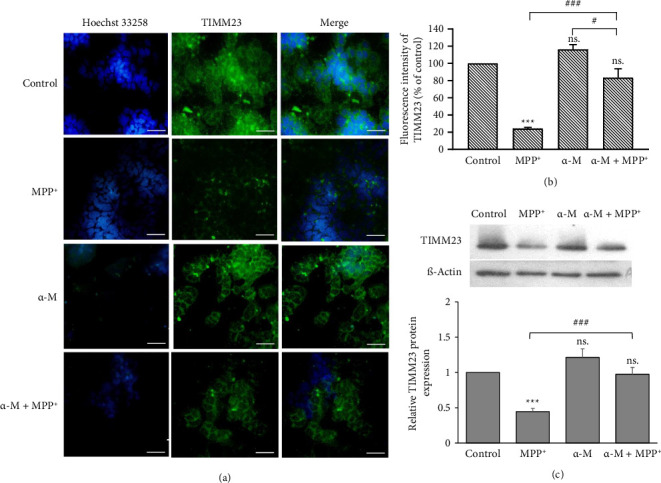
Expression of mitochondrial protein TIMM23 in MPP^+^-treated differentiated SH-SY5Y cells pretreated with α-M. (a) Immunofluorescence images of TIMM23 (green). Scale bar = 50 μm. (b) Quantification of TIMM23 immunofluorescence intensity. Values represent the mean percentage of the control ± SEM (*n* = 3). (c) Western blotting analysis and quantification of TIMM23. Expression values were normalized to the control group (set as 1). Data were expressed as mean ± SEM (*n* = 3). ^∗∗∗^*p* < 0.001; ns., not significant compared with the control group. ^#^*p* < 0.05; ^###^*p* < 0.001.

To evaluate defective mitochondria, which could lead to mitophagy, we examined the expression of complex I protein NDUFS3 and inner membrane protein TIMM23. MPP^+^ significantly reduced NDUFS3 immunofluorescence signals (*p* < 0.001), whereas α-M demonstrated no significant changes compared with the control (Figures 5(a) and 5(b)). However, α-M pretreatment followed by MPP^+^ significantly elevated NDUFS3 immunofluorescence signals compared with MPP^+^ alone (*p* < 0.001). These findings were in accordance with the results obtained from the densitometric analysis of Western blot analysis (Figure 5(c)). MPP^+^ significantly decreased NDUFS3 levels (*p* < 0.001), while α-M exhibited no effect compared with the control. Nonetheless, α-M pretreatment followed by MPP^+^ significantly increased NDUFS3 levels compared with MPP^+^ alone (*p* < 0.05). A similar pattern of expression was observed in the immunofluorescence intensity of TIMM23 (Figures 6(a) and 6(b)). MPP^+^ significantly reduced TIMM23 immunofluorescence signals (*p* < 0.001), while α-M exhibited a slight increase compared with the control. However, α-M pretreatment followed by MPP^+^ significantly elevated TIMM23 immunofluorescence signals compared with MPP^+^ alone (*p* < 0.001). Western blot analysis also demonstrated a significant increase of TIMM23 in the MPP^+^ group with α-M pretreatment, compared with MPP^+^ alone (*p* < 0.001; Figure 6(c)).

### 3.6. Effects of α-M Pretreatment on Mitochondrial Mass and Membrane Potential

**Figure 7:**
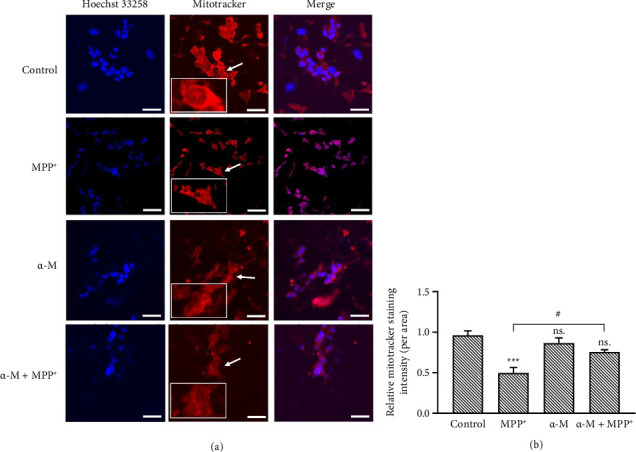
Effects of α-M pretreatment on mitochondrial mass and morphology. (a) Immunofluorescence images of MitoTracker Red. White arrows indicate the rounded and fragmented appearance of mitochondria (magnified in insets). Scale bar = 50 μm. (b) Quantification of MitoTracker Red intensity. Data were expressed as mean ± SEM (*n* = 3). ^∗∗∗^*p* < 0.001; ns., not significant compared with the control group. ^#^*p* < 0.05.

We finally assessed mitochondrial morphology, which could result from the degradation of mitochondrial inner proteins NDUFS3 and TIMM23. Mitochondria were stained with MitoTracker Red and visualized under a fluorescence microscope. In cells treated with MPP^+^, the intensity of mitochondrial staining per area markedly decreased compared with the control, suggesting a decrease in mitochondrial mass. In contrast, the intensity in cells treated with α-M was similar to that in the control group (Figures 7(a) and 7(b)). However, α-M pretreatment followed by MPP^+^ significantly increased fluorescence intensity compared with MPP^+^ alone (*p* < 0.05). The decreased MitoTracker Red intensity observed in the MPP^+^ group was correlated with abnormal mitochondrial morphology, displaying a rounded and fragmented appearance (Figure 6(a)), while cells with α-M pretreatment followed by MPP^+^ showed a lower occurrence of these appearances.

**Figure 8:**
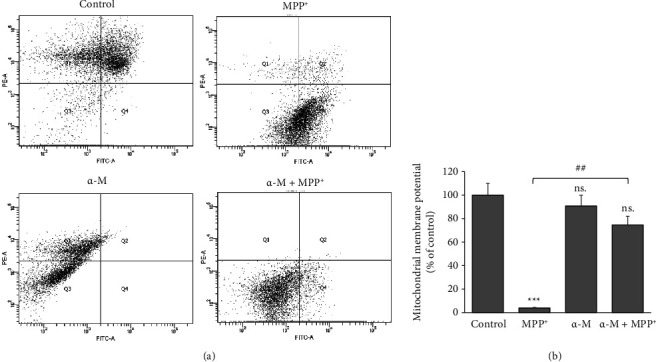
Effects of α-M pretreatment on the mitochondrial membrane potential (ΔΨm) in MPP^+^-treated differentiated SH-SY5Y cells. The ΔΨm was examined using the fluorescent JC-10 probe and analyzed with a flow cytometer. (a) The scatter plots show the quantity of cells emitting light of JC-10 reversible dye. Q1 indicates the quantity of cells emitting light of JC-10 in its monomer form, and Q4 indicates the quantity of cells emitting light of JC-10 in its aggregated form. (b) The bar graphs show the quantification of cells emitting light of JC-10 in the monomer form as a percentage of the control. The data are expressed as the means ± SD from three independent experiments. ^∗∗∗^*p* < 0.001; ns., not significant compared with the control group. ^##^*p* < 0.01.

Since the inner mitochondrial membrane plays a crucial role in maintaining the transmembrane ion gradient, the ΔΨm was examined to evaluate the effect of α-M in MPP^+^-treated cells. This was assessed using the fluorescent JC-10 probe, which penetrates the mitochondrial membrane and accumulates in the matrix in a membrane potential-dependent manner. Flow cytometry analysis of the JC-10 probe showed that SH-SY5Y cells exposed to MPP^+^ exhibited a significant decrease in the ΔΨm (*p* < 0.001) (Figures 8(a) and 8(b)). Treatment with α-M alone did not affect the ΔΨm compared with that in the control. However, pretreatment with α-M prior to MPP^+^ exposure significantly preserved the ΔΨm compared with MPP^+^ treatment alone (*p* < 0.01).

## 4. Discussion

**Figure 9:**
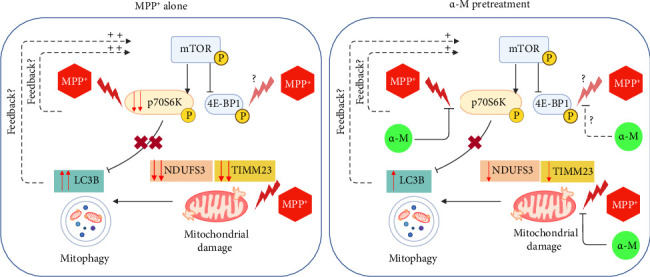
Conceptualized illustration showing the molecular mechanisms through which α-M counteracts MPP^+^-induced mitochondrial damage and dysregulation of mitophagy.

The study highlights the neuroprotective effects of α-M in an MPP^+^-induced PD model using SH-SY5Y neuronal cells. Differentiation of SH-SY5Y cells into mature dopaminergic neurons was confirmed by the increased expression of TH. MPP^+^, a known inducer of mitochondrial dysfunction, significantly decreased the expression of the mitochondrial proteins NDUFS3 and TIMM23, leading to mitochondrial damage. This mitochondrial injury subsequently triggers mitophagy, as evidenced by an increase of LC3B, a key autophagy marker. The mTOR signaling pathway is also implicated, with MPP^+^ affecting its downstream targets, such as p70S6K and 4E-BP1, although the precise effect on 4E-BP1 remains unclear. While MPP^+^ causes mitochondrial damage, our study showed that α-M mitigates these effects, as demonstrated by restoring NDUFS3 and TIMM23 levels, as well as preserving the ΔΨm, suggesting a protective role in preserving mitochondrial integrity. α-M appears to exert its neuroprotective effects through multiple mechanisms. One notable effect is its potential counteraction with MPP^+^-induced suppression of p70S6K, which could restore balance in mTOR signaling. Additionally, α-M pretreatment restores the conversion of LC3-I to LC3-II and the expression of Beclin-1, indicating diminished mitophagy as a result of decreased mitochondrial damage.  Figure 9 illustrates the molecular mechanisms through which α-M counteracts MPP^+^-induced mitochondrial damage.

The findings are consistent with prior studies, including our own, which demonstrate that MPP^+^ exposure reduces NDUFS3 and TIMM23 levels in SH-SY5Y dopaminergic neurons [16, 31, 32]. The loss of these proteins, which reflects impaired mitochondrial protein import and respiratory chain complex I function, is associated with the activation of autophagy [33, 34]. In addition, the PINK1-Parkin pathway plays a crucial role in removing damaged mitochondria. When mitochondria are damaged, the protein PINK1 accumulates on the outer mitochondrial membrane (OMM), where it stabilizes and recruits Parkin, an E3 ubiquitin ligase. Parkin then ubiquitinates proteins on the OMM, marking the damaged mitochondria for degradation via mitophagy [35]. Importantly, α-M alone did not alter NDUFS3 and TIMM23 expression or cell viability across concentrations ranging from 0.25 to 10 μM, indicating it is nontoxic and does not interfere with normal physiological conditions. However, when used as a pretreatment, α-M demonstrates significant protective effects against MPP^+^-induced mitochondrial damage. Our previous study has shown that 10-μM α-M alone does not increase ROS production or affect markers of apoptosis, such as Bax/Bcl-2, p53, or cleaved caspase-3 expression, nor does it increase apoptotic cell counts [26]. Similarly, SH-SY5Y cells treated with 1 μM α-M for 3 h exhibited no signs of toxicity [23]. These findings suggest that α-M is not inherently toxic at concentrations less than 10 μM under normal conditions. Instead, its protective effects are likely context-dependent, becoming apparent only under cellular stress.

mTOR, a central regulator of cellular activities such as protein turnover, mitochondrial biogenesis, and mitophagy, acts through its downstream effectors, p70S6K and 4E-BP1 [36]. In translation initiation, mTOR promotes p70S6K phosphorylation and activation while phosphorylating and inactivating 4E-BP1. In this study, MPP^+^ treatment significantly suppressed p70S6K phosphorylation while promoting p-mTOR. These findings align with previous reports demonstrating that MPP^+^ reduces p70S6K phosphorylation in SH-SY5Y cells and activates mTOR in differentiated SH-SY5Y neurons under 1000 μM MPP^+^ for 24-h treatment [37, 38]. These findings suggest that MPP^+^ directly targets downstream effectors of mTOR. Interestingly, α-M treatment promoted the phosphorylation of p70S6K, restored the phosphorylation level of p70S6K in MPP^+^-treated cells, and alleviated the increase in p-mTOR. A similar trend was observed for the phosphorylation of upstream Akt, though the changes were not statistically significant. These results suggest that α-M counteracts MPP^+^ by targeting the downstream effectors of the Akt/mTOR signaling pathway.

Increased levels of p-p70S6K and p-4EBP1, which lead to autophagy inhibition, have been reported in various conditions, including neurotoxicity and neurodegeneration [37, 39, 40]. Conversely, decreased p70S6K levels, accompanied by increased autophagy, have been observed in PD cellular and mouse models [41–43]. In line with these findings, our study showed that MPP^+^-induced suppression of p-p70S6K removes autophagic inhibition, leading to excessive autophagy. This effect, however, is mitigated by α-M. The reduction in MPP^+^-triggered autophagy observed in our study may be attributed to the protective effect of α-M on the mitochondria and p70S6K activity. Specifically, α-M pretreatment was shown to preserve mitochondrial inner membrane proteins, such as NDUFS3 and TIMM23, and maintain mitochondrial morphology. This preservation of mitochondrial integrity reduces the extent of autophagy activation triggered by mitochondrial damage.

The observed increase in p-mTOR during MPP^+^ treatment may reflect a compensatory feedback mechanism to counteract reduced p70S6K activity and excessive autophagy. Excessive autophagy can be detrimental, leading to neuronal death in conditions such as PD [44–47]. Although the precise mechanisms remain unclear, previous evidence suggests that the end products of autophagy might serve as a negative-feedback regulator of mTOR signaling [48]. Evidence in human non-neuronal cells has shown that mTOR interacts with components of the autophagy initiation complex, such as unc-51-like autophagy activating kinase 1 (ULK1) and autophagy-related 13 (Atg13), to inhibit autophagy [49, 50]. However, these feedback mechanisms and their roles in neurotoxin-induced cell death warrant further investigation. Our study suggests that exposure to MPP^+^-induced stress triggers excessive autophagy, making neurons more susceptible to cell death. To counter this, cells activate mTOR as a survival mechanism to suppress autophagy. α-M appears to play a key role in modulating this adaptive response, helping maintain autophagy at a balanced level under conditions of MPP^+^-induced stress.

The effects of α-M on the central nervous system (CNS) are multifaceted, involving both direct neuroprotective actions and indirect modulation through its anti-inflammatory and antioxidant properties [28]. Although its limited ability to cross the blood–brain barrier poses a challenge to its efficacy, recent advancements in drug delivery systems have improved its CNS bioavailability by overcoming this barrier.

## 5. Conclusion

Our study highlights the neuroprotective potential of α-M in mitigating MPP^+^-induced mitochondrial damage and autophagy in dopaminergic neurons. α-M preserves mitochondrial integrity by preventing the degradation of key mitochondrial proteins, such as NDUFS3 and TIMM23, and restores p-p70S6K levels, thereby reducing the excessive autophagy triggered by MPP^+^. These findings suggest that α-M can modulate critical signaling pathways, including Akt/mTOR/p70S6K, to maintain autophagy at a balanced level and protect neurons under oxidative stress conditions. Given these properties, α-M holds promise as a therapeutic candidate for neurodegenerative diseases like PD, where mitochondrial dysfunction and dysregulated autophagy play a pivotal role. Future studies should explore the detailed molecular mechanisms underlying the effects of α-M on mTOR signaling and its interaction with other autophagy regulators, such as ULK1 and Atg13.

## Data Availability

The data that support the findings of this study are available on request from the corresponding author.

## References

[1] Raza C., Anjum R., Shakeel N. U. A. (2019). Parkinson’s disease: Mechanisms, Translational Models and Management Strategies. *Life Sciences*.

[2] Kalia L. V., Lang A. E. (2015). Parkinson’s disease. *The Lancet*.

[3] Mattson M. P. (2000). Apoptosis in Neurodegenerative Disorders. *Nature Reviews Molecular Cell Biology*.

[4] Kotake Y., Ohta S. (2003). MPP^+^ Analogs Acting on Mitochondria and Inducing Neuro-Degeneration. *Current Medicinal Chemistry*.

[5] Lopert P., Patel M. (2016). Mitochondrial Mechanisms of Redox Cycling Agents Implicated in Parkinson’s disease. *Journal of Neural Transmission*.

[6] Cassarino D. S., Parks J. K., Parker W. D., Bennett J. P. (1999). The Parkinsonian Neurotoxin MPP^+^ Opens the Mitochondrial Permeability Transition Pore and Releases Cytochrome C in Isolated Mitochondria via an Oxidative Mechanism. *Biochimica et Biophysica Acta (BBA)—Molecular Basis of Disease*.

[7] Tatton W. G., Chalmers-Redman R., Brown D., Tatton N. (2003). Apoptosis in Parkinson’s disease: Signals for Neuronal Degradation. *Annals of Neurology*.

[8] Li J., Yin K., Hou L. (2023). Polystyrene Microplastics Mediate Inflammatory Responses in the Chicken Thymus by Nrf2/NF-κB Pathway and Trigger Autophagy and Apoptosis. *Environmental Toxicology and Pharmacology*.

[9] Ramalingam M., Kim S. J. (2016). Insulin on Activation of Autophagy With Integrins and Syndecans Against MPP(+)-Induced Alpha-Synuclein Neurotoxicity. *Neuroscience Letters*.

[10] Cuervo A. M. (2004). Autophagy: Many Paths to the Same End. *Molecular and Cellular Biochemistry*.

[11] Friedman L. G., Lachenmayer M. L., Wang J. (2012). Disrupted Autophagy Leads to Dopaminergic Axon and Dendrite Degeneration and Promotes Presynaptic Accumulation of Alpha-Synuclein and LRRK2 in the Brain. *Journal of Neuroscience*.

[12] Kim Y. C., Guan K. L. (2015). mTOR: A Pharmacologic Target for Autophagy Regulation. *Journal of Clinical Investigation*.

[13] Lashgari N. A., Roudsari N. M., Momtaz S., Sathyapalan T., Abdolghaffari A. H., Sahebkar A. (2021). The Involvement of JAK/STAT Signaling Pathway in the Treatment of Parkinson’s disease. *Journal of Neuroimmunology*.

[14] Zhao X. H., Wang Y. B., Yang J., Liu H. Q., Wang L. L. (2019). MicroRNA-326 Suppresses Inos Expression and Promotes Autophagy of Dopaminergic Neurons Through the JNK Signaling by Targeting XBP1 in a Mouse Model of Parkinson’s Disease. *Journal of Cellular Biochemistry*.

[15] Wang R., Hou L., Lu H. (2024). Unveiling the Interplay of MAPK/NF-κB/MLKL Axis in Brain Health: Omega-3 as a Promising Candidates Against Copper Neurotoxicity. *Journal of Environmental Management*.

[16] Chanthammachat P., Dharmasaroja P. (2019). Metformin Restores the Mitochondrial Membrane Potentials in Association With a Reduction in TIMM23 and NDUFS3 in MPP^+^-Induced Neurotoxicity in SH-SY5Y Cells. *EXCLI Journal*.

[17] Demishtein-Zohary K., Azem A. (2017). The TIM23 Mitochondrial Protein Import Complex: Function and Dysfunction. *Cell and Tissue Research*.

[18] Franco-Iborra S., Cuadros T., Parent A., Romero-Gimenez J., Vila M., Perier C. (2018). Defective Mitochondrial Protein Import Contributes to Complex I-Induced Mitochondrial Dysfunction and Neurodegeneration in Parkinson’s disease. *Cell Death & Disease*.

[19] Cheng J. H., Huang A. M., Hour T. C., Yang S. C., Pu Y. S., Lin C. N. (2011). Antioxidant Xanthone Derivatives Induce Cell Cycle Arrest and Apoptosis and Enhance Cell Death Induced by Cisplatin in NTUB1 Cells Associated With ROS. *European Journal of Medicinal Chemistry*.

[20] Zhao Y., Liu J. P., Lu D., Li P. Y., Zhang L. X. (2010). A New Antioxidant Xanthone From the Pericarp of *Garcinia mangostana* Linn. *Natural Product Research*.

[21] Kondo M., Zhang L., Ji H., Kou Y., Ou B. (2009). Bioavailability and Antioxidant Effects of a Xanthone-Rich Mangosteen (*Garcinia mangostana*) Product in Humans. *Journal of Agricultural and Food Chemistry*.

[22] Alam M., Rashid S., Fatima K. (2023). Biochemical Features and Therapeutic Potential of Alpha-Mangostin: Mechanism of Action, Medicinal Values, and Health Benefits. *Biomedicine & Pharmacotherapy*.

[23] Ruankham W., Suwanjang W., Phopin K., Songtawee N., Prachayasittikul V., Prachayasittikul S. (2022). Modulatory Effects of Alpha-Mangostin Mediated by SIRT1/3-FOXO3a Pathway in Oxidative Stress-Induced Neuronal Cells. *Frontiers in Nutrition*.

[24] Ahmadian R., Heidari M. R., Razavi B. M., Hosseinzadeh H. (2023). Alpha-Mangostin Protects PC12 Cells Against Neurotoxicity Induced by Cadmium and Arsenic. *Biological Trace Element Research*.

[25] Ghobakhlou F., Eisvand F., Razavi B. M., Ghasemzadeh Rahbardar M., Hosseinzadeh H. (2023). Evaluating the Effect of Alpha-Mangostin on Neural Toxicity Induced by Acrylamide in Rats. *Environmental Science and Pollution Research*.

[26] Janhom P., Dharmasaroja P. (2015). Neuroprotective Effects of Alpha-Mangostin on MPP(+)-Induced Apoptotic Cell Death in Neuroblastoma SH-SY5Y Cells. *Journal of Toxicology*.

[27] Hao X. M., Li L. D., Duan C. L., Li Y. J. (2017). Neuroprotective Effect of Alpha-Mangostin on Mitochondrial Dysfunction and Alpha-Synuclein Aggregation in Rotenone-Induced Model of Parkinson’s disease in Differentiated SH-SY5Y Cells. *Journal of Asian Natural Products Research*.

[28] Hu Z., Wang W., Ling J., Jiang C. (2016). α-Mangostin Inhibits α-Synuclein-Induced Microglial Neuroinflammation and Neurotoxicity. *Cellular and Molecular Neurobiology*.

[29] Parkhe A., Parekh P., Nalla L. V. (2020). Protective Effect of Alpha Mangostin on Rotenone Induced Toxicity in Rat Model of Parkinson’s disease. *Neuroscience Letters*.

[30] Parekh P., Sharma N., Sharma M. (2022). AMPK-Dependent Autophagy Activation and Alpha-Synuclein Clearance: A Putative Mechanism Behind Alpha-Mangostin’s Neuroprotection in a Rotenone-Induced Mouse Model of Parkinson’s Disease. *Metabolic Brain Disease*.

[31] Dutta D., Ali N., Banerjee E. (2018). Low Levels of Prohibitin in Substantia Nigra Makes Dopaminergic Neurons Vulnerable in Parkinson’s Disease. *Molecular Neurobiology*.

[32] Parsons R. B., Aravindan S., Kadampeswaran A. (2011). The Expression of Nicotinamide N-Methyltransferase Increases ATP Synthesis and Protects SH-SY5Y Neuroblastoma Cells Against the Toxicity of Complex I Inhibitors. *Biochemical Journal*.

[33] Lin X., Wen X., Wei Z. (2021). Vitamin K2 Protects Against Aβ42-Induced Neurotoxicity by Activating Autophagy and Improving Mitochondrial Function in Drosophila. *NeuroReport*.

[34] Eldeeb M. A., Bayne A. N., Fallahi A. (2024). Tom20 Gates PINK1 Activity and Mediates Its Tethering of the TOM and TIM23 Translocases upon Mitochondrial Stress. *Proceedings of the National Academy of Sciences of the United States of America*.

[35] Wang S., Long H., Hou L. (2023). The Mitophagy Pathway and Its Implications in Human Diseases. *Signal Transduction and Targeted Therapy*.

[36] de la Cruz Lopez K. G., Toledo Guzman M. E., Sanchez E. O., Garcia Carranca A. (2019). mTORC1 as a Regulator of Mitochondrial Functions and a Therapeutic Target in Cancer. *Frontiers in Oncology*.

[37] Xie Q., Liu M., Yan Y. F., Shen X., Wang E. S. (2019). Exogenous Tetranectin Protects Against 1-Methyl-4-Phenylpyridine-Induced Neurotoxicity by Inhibiting Apoptosis and Autophagy Through Ribosomal Protein S6 Kinase Beta-1. *World Neurosurgery*.

[38] Khwanraj K., Prommahom A., Dharmasaroja P. (2023). eEF1A2 Sirna Suppresses MPP(+)-Induced Activation of Akt and Mtor and Potentiates Caspase-3 Activation in a Parkinson’s disease Model. *The Scientific World Journal*.

[39] Wei R., Zhang X., Cai Y. (2020). Busulfan Suppresses Autophagy in Mouse Spermatogonial Progenitor Cells via mTOR of AKT and p53 Signaling Pathways. *Stem Cell Reviews and Reports*.

[40] Yin Y., Dang W., Zhou X. (2018). PI3K-Akt-mTOR Axis Sustains Rotavirus Infection via the 4E-BP1 Mediated Autophagy Pathway and Represents an Antiviral Target. *Virulence*.

[41] Pan Y., Chen M., Pan L. (2024). Shisandra Decoction Alleviates Parkinson’s Disease Symptoms in a Mouse Model Through PI3K/AKT/mTOR Signalling Pathway. *Neuropsychiatric Disease and Treatment*.

[42] Wang X., Hu W., Qu L. (2023). Tricin Promoted ATG-7 Dependent Autophagic Degradation of Alpha-Synuclein and Dopamine Release for Improving Cognitive and Motor Deficits in Parkinson’s disease. *Pharmacological Research*.

[43] Jayaraj R. L., Beiram R., Azimullah S. (2021). Noscapine Prevents Rotenone-Induced Neurotoxicity: Involvement of Oxidative Stress, Neuroinflammation and Autophagy Pathways. *Molecules*.

[44] Gao X., He D., Liu Y. (2023). Oral Administration of Limonin (LM) Exerts Neuroprotective Effects by Inhibiting Neuron Autophagy and Microglial Activation in 6-OHDA-Injected Rats. *International Immunopharmacology*.

[45] Zhou L., Cheng Y. (2019). Alpha-Lipoic Acid Alleviated 6-OHDA-Induced Cell Damage by Inhibiting AMPK/mTOR Mediated Autophagy. *Neuropharmacology*.

[46] Su Y. C., Qi X. (2013). Inhibition of Excessive Mitochondrial Fission Reduced Aberrant Autophagy and Neuronal Damage Caused by LRRK2 G2019S Mutation. *Human Molecular Genetics*.

[47] Deneubourg C., Ramm M., Smith L. J. (2022). The Spectrum of Neurodevelopmental, Neuromuscular and Neurodegenerative Disorders Due to Defective Autophagy. *Autophagy*.

[48] Liang C. (2010). Negative Regulation of Autophagy. *Cell Death & Differentiation*.

[49] Chan E. Y., Longatti A., McKnight N. C., Tooze S. A. (2009). Kinase-Inactivated ULK Proteins Inhibit Autophagy via Their Conserved C-Terminal Domains Using an Atg13-Independent Mechanism. *Molecular and Cellular Biology*.

[50] Hara T., Takamura A., Kishi C. (2008). FIP200, A ULK-Interacting Protein, is Required for Autophagosome Formation in Mammalian Cells. *The Journal of Cell Biology*.

